# The role of exercise in modifying outcomes for people with multiple sclerosis: a randomized trial

**DOI:** 10.1186/1471-2377-13-69

**Published:** 2013-06-28

**Authors:** Nancy E Mayo, Mark Bayley, Pierre Duquette, Yves Lapierre, Ross Anderson, Susan Bartlett

**Affiliations:** 1 Division of Clinical Epidemiology, Royal Victoria Hospital, McGill University Health Centre, Montreal, QC H3A 1A1, Canada; 2 UHN - Toronto Rehabilitation Institute, University Centre, 550 University Avenue, Toronto, ON M5G 2A2, Canada; 3 Centre hospitalier de l’Université de Montréal, 1560 Sherbrooke Street E, Montreal, Quebec H2L 4M1, Canada; 4 Montreal Neurological Institute and Hospital, 3801 University Street, Montreal, Quebec H3A 2B4, Canada; 5 Department of Kinesiology and Physical Education, Faculty of Education, McGill University, Montreal, QC H2W 1S4, Canada

## Abstract

**Background:**

Despite the commonly known benefits of exercise and physical activity evidence shows that persons Multiple Sclerosis (MS) are relatively inactive yet physical activity may be even more important in a population facing functional deterioration. No exercise is effective if it is not done and people with MS face unique barriers to exercise engagement which need to be overcome. We have developed and pilot tested a Multiple Sclerosis Tailored Exercise Program (MSTEP) and it is ready to be tested against general guidelines for superiority and ultimately for its impact on MS relevant outcomes. The primary research question is to what extent does an MS Tailored Exercise Program (MSTEP) result in greater improvements in exercise capacity and related outcomes over a one year period in comparison to a program based on general guidelines for exercise among people with MS who are sedentary and wish to engage in exercise as part of MS self-management.

**Methods/Design:**

The proposed study is an assessor-blind, parallel-group, randomized controlled trial (RCT). The duration of the intervention will be one year with follow-up to year two. The targeted outcomes are exercise capacity, functional ambulation, strength, and components of quality of life including frequency and intensity of fatigue symptoms, mood, global physical function, health perception, and objective measures of activity level. Logistic regression will be used to test the main hypothesis related to the superiority of the MSTEP program based on a greater proportion of people making a clinically relevant gain in exercise capacity at 1 year and at 2 years, using an intention-to-treat approach. Sample size will be 240 (120 per group).

**Discussion:**

The MS community is clearly looking for interventions to help alleviate the disabling sequelae of MS and promote health. Exercise is a well-known intervention which has known benefits to all, yet few exercise regularly. For people with MS, the role of exercise in MS management needs to be rigorously assessed to inform people as to how best to use exercise to reduce disability and promote health.

**Trial registration:**

Clinical Trials.gov: NCT01611987

## Background

Multiple Sclerosis (MS) is a progressive and chronic disease affecting many North American young adults who are at the peak of their career and family development [1-6]. Persons with MS commonly report problems with walking, balance, fatigue and visual disturbances [5,6]. These symptoms can appear suddenly, they have a variable course and they differ in severity. They all, however, progress with age and ultimately can have a devastating impact on the health and quality of life [7-9].

The cause of MS is unknown and the cure is yet to be found. Technological advances such as the MRI, however, have made early diagnosis possible. New medications slow disease progression, usually only in the relapsing-remitting variant of the disease not the primary progressive or secondary progressive forms [6,10,11]. The full benefits of advances in diagnosis and treatment will not be realized unless people with MS are encouraged to develop and maintain a level of physical conditioning that will allow them to live full lives. In addition, there is growing consensus that exercise could exert an immunomodulation role which could be neuroprotective [12-14]. Therefore, some therapeutic exercise could be neurorehabilitative, as with people with stroke [15].

Despite the commonly known benefits of exercise and physical activity participation [16-18], evidence shows that persons with MS participate in physical activity at a level more than ½ standard deviation below that of a non-MS population (effect size -0.60; 95% CI: -0.44 to -0.77) [19]. Hence, persons with MS (predominantly women) may adopt a sedentary lifestyle with the added risk of developing secondary health conditions such as heart disease, osteoporosis, obesity, and diabetes. The role of exercise and physical activity may be even more important in a population facing functional deterioration [14,19,20].

We recently updated [21] a 2004 Cochrane Review [22] and summarized the results 11 randomized trials on exercise with a combined total of 502 persons with MS. The focus of this review was on evidence for prescribing exercise and the conclusion was that, due to the broad range of exercise interventions, it is not possible to make unified exercise recommendation as to what type of exercise is safe and effective for persons with MS. The studies covered four main types of exercise (i) aerobic exercise (walking, bicycling, and aquatic exercise); (ii) yoga; (iii) resistance exercise; or (iv) stretching. The duration of the interventions ranged from 3 weeks to 6 months, lasted 30 to 60 minutes per session, and the intervention frequency ranged from one to five times a week with different levels of intensity. Only one of the 11 studies reported an effect size with a 95% confidence interval excluding the null value of no difference between groups. Literature published subsequent to our 2009 review [23-31] has not clarified the role of exercise in MS. A recent (2011) study by Collet [27] tested 3 different exercise intensities of cycling exercise on change in walking capacity. There was no significant gain in walking capacity for the group (n = 20) assigned to continuous cycling at 45% peak power. The group assigned to 30 sec. on and 30 sec. off at 90% peak power (n = 18) showed an increase in distance walked in 2 minutes (2MWT) of 13 m (95% CI 4 to 22); the group receiving a combination of these two approaches (n = 17) showed no significant gain on 2MWT. This research supports the use of high intensity interval training for people with MS. There was a drop off in attendance after the 6 week program and no further gains were made. The intermittent group with alternating high intensity cycling and rest experienced some leg pain when cycling.

However, there is evidence for the effectiveness of various components of exercise in MS [23,25,32,33]. Two studies [32,33] demonstrated that aerobic exercise in comparison to no aerobic exercise increased VO_2_ peak and related parameters. Three studies clearly demonstrated the effectiveness of strength training [23], endurance training [25], and power training [34]. There are new studies [35-38] showing that pelvic floor exercises, part of the core musculature, are effective in improving urinary incontinence for women.

At the moment, there is insufficient evidence for prescribing comprehensive exercise programs for people with MS [21] either for functional improvement or health promotion, let alone for immunomodulation. However, several groups have made general recommendations for exercise [14,18,22,39-42]. The most recent guidelines were produced in 2012 [43] and recommend 30 minutes of moderate intensity aerobic activity, 2 times per week, and strength training exercises for major muscle group, 2 times per week.

The MS Societies of Canada, United States, Great Britain and Australia all provide documentation about the benefits of exercise for people with MS. But exercise is not effective if people will not do it.

We recently surveyed 417 persons with MS [44] and found that 40% were not exercising on a regular basis; not vastly different some reports from surveys of the general population [45]. The top three barriers to exercise were *being too tired (86%)*, *having MS related impairments (61%)*, and *lack of time* (58%). Barriers such as *lack of interest, lack of information regarding exercise recommendation*, *interference with other responsibilities*; *feeling I can’t do things correctly*; *dislike exercise*; and *find exercise boring,* were more commonly endorsed by non-exercisers than exercisers. This suggests that if non-exercisers are to be engaged in regular exercise, the program must be interesting, relevant to the individual, and easily implemented across a variety of settings. However what was missing for people with MS that is different for people without MS is that people with MS expressed the need to feel safe and have clear professional instructions provided on proper techniques, intensity and duration.

In addition, our recently completed study of the life impact of MS [46] showed, for a sample of 185 persons diagnosed since 1994, that static balance, physical function and functional walking capacity (six minute walk test) were within 70% of norms for age and sex, but individuals in the sample were < 50%ile for central core strength, grip strength, and muscle power, and < 25%ile for upper body core strength and exercise capacity (i.e., peak V0_2_). In addition, almost ¼ of the sample had spasticity that would complicate exercise prescription requiring an adapted exercise program. These findings suggest that in people with MS, although some have generalized weakness and deconditioning, the majority have specific problems that require tailored exercise solutions.

### Objectives

The global aim of this study is to contribute evidence for the role of targeted exercise in altering MS outcomes over time. The primary research question is to what extent does an MS Tailored Exercise Program (MSTEP) result in greater improvements in exercise capacity and related outcomes over a one year period in comparison to a program based on general guidelines for exercise among people with MS who are sedentary and wish to engage in exercise as part of MS self-management. The primary outcome for this question is exercise capacity measured using cycle ergometry. However exercise efficiency, functional ambulation, strength, components of quality of life including frequency and intensity of fatigue symptoms, mood, global physical function, and health perception will also be measured as components of a global response outcome. The first confirmatory hypothesis is that MSTEP will result in a greater proportion of people making clinically relevant gains (at least 10% change) in exercise capacity than with general guidelines after 12 months of intervention; a secondary hypothesis is that, while there may be some decline in exercise capacity among individuals from end of intervention to follow-up one year later, the decline will be greater in the general guideline group augmenting the difference between groups in the proportion making 10% change from study entry to 24 months. In other words, gains will be maintained more for the MSTEP group over the general guideline group.

An exploratory hypothesis is that more of the targeted outcomes will improve with the MSTEP program than the general guideline approach. An explanatory hypothesis is that these gains will be accompanied by reports of greater exercise self-efficacy (confidence) with the MSTEP program than with the general guideline program leading to more consistent exercise engagement and improved long-term adherence.

## Methods

### Trial design

**Figure 1:**
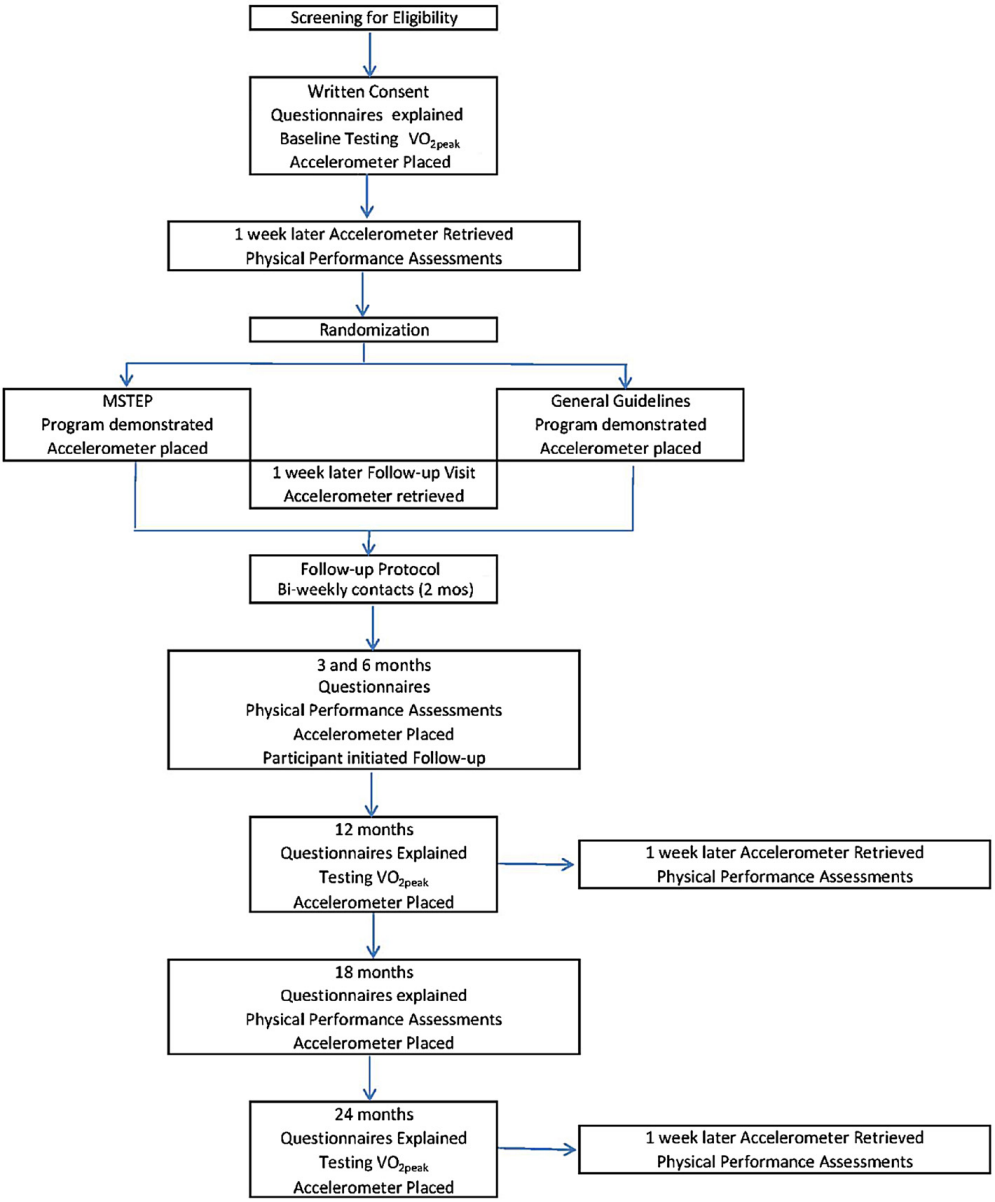
The path of participants through the study protocol.

The proposal is for a two-group, assessor-blind, stratified, randomized, pragmatic, trial. Those consenting will be randomly assigned with a 1:1 ratio to either the MSTEP program or the general exercise guideline program. The intervention period will be one year with follow-up to a second year. The path of study participants through the study protocol is shown in Figure 1. The trial is registered with ClinicalTrials.gov NCT01611987. The trial received ethical approval from the Montreal Neurological Hospital Research Ethics Board (NEU-12-005). Written informed consent will be obtained from all participants in the study.

### Participants

The target population is community dwelling, adults (aged 19 to 65) with MS diagnosed after 1994. Specific inclusion criteria are: (i) ambulatory (can walk 100 m. be capable of walking 100 meters without a walking aid (EDSS ≤ 5.5), even if they do use an aid for daily activities; and (ii) sedentary or irregularly active at time of study entry (i.e. do not exercise 30 minutes or more twice per week of moderate to vigorous activity).

Excluded will be people who are (i) unable to speak and read English or French; (ii) unable to respond to simple questions on orientation and memory; (iii) have an additional illness that restricts their function; and/or (iv) had suffered at least one relapse during the past 30 days (as defined by Polman in 2011 [47]) as this may affect physical activity/exercise participation. Potential participants will be identified from the population of persons enrolled in three MS clinics in the Montreal area and in three clinics in Toronto.

### Interventions

#### MSTEP

The Multiple Sclerosis Tailored Exercise Program (MSTEP) was developed based on the results of our systematic review [21], results of several studies conducted by our group on the health outcomes and exercise preferences in people with MS [44,46], pilot experience, knowledge from both physical therapy practice and exercise training, and input from patients.

MSTEP was designed to meet the exercise and disability needs of people with MS, avoid fatigue or heat exhaustion, as well as to offer the convenience of being able to incorporate exercise into a participant’s daily routine (e.g. performed on their way to work or at a lunch break). MSTEP provides the person with MS the opportunity to be taught a program informed by exercise science and physical therapy including how to safely execute and adapt a variety of exercises targeting endurance, muscular and core strength, balance, flexibility, muscular power, and speed of movement to their needs. The goal of the MSTEP program is to promote regular bouts of activity most days per week, encouraging a balance between rest and activity, and taking into consideration the physical and emotional status and capacity of the person which fluctuates from day to day in MS.

There are five primary components to the program: (i) cardio-aerobic/endurance; (ii) core strength; (iii) peripheral muscle strength; (iv) power; and (v) flexibility.

(i) Cardio aerobic/endurance will be trained using two types of activities. Cardio-intensive exercise, prescribed, two times per week will use interval training. We are calling these days “Push-Days” and have specifically included interval training as it has been shown it is safe [48] and can be more effective than continuous endurance training, even in people with chronic health conditions [27,49]. In addition, this type of training empowers individuals to balance rest, activity, and control (select) optimal days to work at a high exercise intensity level. On Push Days, participants will do a short bursts of moderate-high intensity exercise (e.g., for 1 to 3 minutes) then reduce to a more comfortable pace for 5–7 minutes. During demonstration with the exercise instructor, the intensity of the interval training will be monitored by a portable heart rate monitor; participants will be taught how to use the Borg scale with target of 16 (Very hard) followed by reduced intensity that is comfortable (11-very light) [50]. Push-days offer a way for subjects to “step-up” the intensity of the program as a way of increasing power, strength and endurance. The on/off intensity intervals will be self-selected working up to 1-on/1-off over the one year period; a recent study used supervised 30 sec on/30 sec off exercise but the participants experienced leg pain and but did not sustain this when the program ended. We feel our approach of self-selecting the intervals would reduce side effects and provides a challenge for the person lengthen the on-interval and shorten the off-interval as they increase their exercise capacity. This activity is done for a minimum of 10 minutes with progression as tolerated.

The second activity, to be done on most other days of the week (eg. 4), is moderate intensity (brisk) walking, and we will suggest that they wear a pedometer to monitor progress, beginning with 10 min and working toward a goal of achieving up to 30 min most days of the week. To increase variety and options for these walks, individuals can chose to carry light weights, follow metronome pacing, or use Nordic Walking Poles (which also assist with balance) or they can choose other forms of exercise such as swimming or biking. On two other non-Push Days, participants will choose from a menu of muscular strength, core strength and balance, or muscular power and speed of movement exercise options, alternating each day.

(ii) Core strength will be trained by using adaptations of exercises commonly taught in courses in Pilates and Yoga. The focus is on muscles of the axial, pelvic, and abdominal regions. Persons will be taught to sue different pieces of equipment such as an exercise ball, roll or dome to recruit core muscles as well as simple core exercises to do without equipment.

Peripheral muscle strength will focus on the major muscle groups with a particular emphasis on anti-gravity muscles. Specific exercises will be taught for muscles that are tested to be weak.

(iii) Power exercises will train strength per unit time and will focus on muscles required for activities done in bursts such as stair climbing, rising from a chair, getting up from the floor, running, and jumping.

(iv) Flexibility exercises will focus at a minimum on calf muscles (particular important for people with spasticity), hamstrings, and shoulder girdle muscles. Additional flexibility exercises will be taught for areas assessed to be on concern (adductors, quadriceps, hip flexors, trunk).

To start each component will be prescribed for 10 minutes with the two cardio-aerobic activities increasing over time as the participant is able targeting 30 minutes per day. The MSTEP targets daily (6/7) activity as disability does not take a holiday. Flexibility is prescribed 6 days per week, 60 moderate aerobic exercise such as brisk walking is prescribed 4 days per week; 120 the other 4 components Push-Day, Core, Strength, Power are prescribed 2 days per week. 60 Thus on anyone day, a person would do flexibility (10 minutes) and 2 other components of the person’s choice. We recommend that on the Push-Day, participants choose a less vigorous activity (eg. Core).

The participants in the MSTEP will have the opportunity to practice on different types of equipment so that they may decide for themselves if they wish to purchase one or more pieces of equipment. They will also be given photo images or drawings of exercises that have been tailored to them. They will also be coached with respect to progression by changing starting position, duration, support, and inclination. Overall, if the person can do the exercise, they need to progress.

MSTEP is designed to provide a tailored approach and part of the intervention is to identify preferred ways of exercising and preferred times and work with the participant to develop a personalized exercise schedule. Participants may wish to join a gym or take a Yoga or Pilates class and this would be an acceptable way of working on flexibility, core strength and strength.

Two other aspects of tailoring will be trained: goal setting and implementation intention. Goal setting will follow the SMART goal approach (Specific, Measureable, Attainable, Realistic, and Time specific) [51,52]. Implementation intention using mental imagery [53,54] to imagine situations when the participant will not be able to meet their exercise goal, develop, write out, and visualize these alternate plans.

#### General guideline approach

The control group will be given the 2012 exercise guidelines for adults with MS from the Canadian Society for Exercise Physiology [43] which recommends 30 minutes of aerobic and strength training two times per week. Aerobic exercise is gradually increased until 30 minutes is reached for each workout session and done at a moderate intensity of 5 or 6 on a scale where 10 is the maximum. At this intensity, the exerciser could talk but not sing. Examples of activities are upper or lower body cycling, walking, elliptical training or aquatic or land exercises.

Strength training could be done on the same or different day from the aerobic exercise as long as there is one day rest between strength training sessions. The guideline is to work up to doing two sets of 10 to 15 repetitions of each exercise with a rest of one to two minutes in between sets of exercises. Resistance can be provided by free weights, cable pulleys, bands, or weight machines. An appropriate weight is one that can be lifted barely but safely 10 to 15 times.

#### Elements common to both groups

Both conditions target key components of physical fitness including aerobic capacity and strength. Exercise progression in both programs is slow and gradual. All individuals will be taught how to take their heart rate and work within the age-recommended range, and use the Borg scale to adjust exercise intensity. The overall goal is increase physical activity over time in a safe and effective manner to levels that have been shown to result in benefits to overall physical fitness and health for adults with chronic illness.

Participants will be trained in the exercise program to which they are assigned. Participants in both conditions will receive two private training sessions with the exercise instructor to assess individual needs and learn how to apply principles of safe and effective exercise. The importance of making modifications to accommodate individual needs and MS in general will be discussed. All will be provided with a portfolio of exercise instructions.

All participants will be contacted every two weeks during the first two months, then monthly thereafter by the exercise instructor by telephone or email for follow-up by the instructor. The purpose of follow-up contacts is to provide an opportunity to ask any questions they have and also to facilitate long-term retention in the study. All will have their exercise regimens reviewed during the intervention year at the evaluations which are scheduled at 3, 6, and 12 months.

### Outcomes

The targeted outcomes are exercise capacity, exercise efficiency, functional ambulation, strength, and components of quality of life including frequency and intensity of fatigue symptoms, mood, global physical function, health perception, and illness intrusiveness. Also collected will be exercise adherence, exercise enjoyment, socio-demographic, adverse events, and clinical information including relapse rate. The complete portfolio of outcomes [55-79] is listed in Table 1.

**Table 1:** Outcomes for MSTEP Study

**Construct**	**Measure**	**Clinically relevant change**
Primary Outcome		
Exercise capacity	V0_2peak_	10% change[32,55,56]
Components of Global Outcome		
Exercise efficiency: Gross, net and work efficiency	Gross efficiency = work performed / energy expended × 100%, Net efficiency = work performed / energy expended above rest × 100%, and Work efficiency = work performed / energy expended above that in cycling at 0 W × 100% [57].	10% change [58].
Sub-maximal exercise capacity	The Modified Canadian Aerobic Fitness Test (mCAFT) is a multi-stage step-test, simple to use and inexpensive, that assesses sub-maximal aerobic capacity [59].	1 stage [59]
Functional ambulation	Modified 6 Minute Walk Test (M-6MWT) [60]; distance and fatigability index calculated by [the distance walked in the last minute ÷ distance walked in the first minute], with ratio ≥ 1.0 indicating less fatigability; Reliability high (ICC across 3 walks, 0.95).	50 m. [32,60-64].
Strength	Grip, vertical jump, push-ups, curl-ups [65]	½ SD: jump 5cm; curl ups 5; push ups 3; grip strength 12 [46].
Fatigue symptoms	Unidimensional Fatigue Impact Scale [66]; which is a Rasch validated measure of fatigue modeled from the original and modified versions [60,67]	Minimum clinically important difference(MCID) 5; ½ SD = 6 [66].
Mood*	Rand-36 MHI subscale [68,69];	Meaningful change: 10
Global physical function*	Rand-36 PF subscale [68,69]	Meaningful change = 10
Health perception*	EQ-5D [70-73]	Meaningful change = 10
Quality of life*	Patient Generated Index* [74,75]	Meaningful change ½ SD = 12.5
Explanatory Variables		
Current disability level	PDDS (patient version of EDSS) for all persons [76]	
Relapses	Defined as “patient-reported or objectively observed events typical of an acute inflammatory demyelinating event in the CNS, current or historical, with duration of at least 24 hours, in the absence of fever or infection.” [47]; annualized rate over study period	
Exercise adherence	Exercise diary (paper, computer version) daily for 3 months, 1 week every 3 months subsequently; accelerometers worn for 1 week every 3 months (see response to reviewers on feasibility of this) ActivPal [77]	
Exercise self-efficacy	3 item questionnaire with demonstrated validity and test-retest reliability (>0.85) [78]	
Exercise barriers and benefits	The benefits subscale of the Exercise Benefits/Barriers Scale will be used [79] along with 4 indicators for barriers shown to be independent predictors of exercise engagement [21,44]	

* Data from ½ SD from PGI and SF-36 subscales from recently completed CIHR pilot grant on Gender differences in Life Impact of MS which is described in the first publication from this data set [46].

### Primary outcome

The first primary effectiveness outcome will be VO_2peak_ using a modified Bruce protocol on the cycle ergometer. Briefly, peak oxygen consumption (VO_2peak_) will be determined using an incremental graded cycle ergometer test. The person will properly seated and the bicycle adjusted for optimally positioning. Rate of Perceived Exertion (RPE), heart rate, and blood pressure will be taken at each workload, in addition to assessing oxygen consumption and CO_2_ production. The person will cycle for 3 minutes at 0 watts, 10 watts and 20 watts (9 minutes) and subsequently, at intervals of 1 minute, the work load will be increased by 10 watts. People will be considered to have reached their peak exertion if one of the following criteria are met [80]: (i) reached their age-predicted heart rate of 220-age; (ii) a rating of perceived exertion of at least 17 on the Borg scale; (iii) a respiratory rate of 35 breaths per minute; (iv) pedalling rate of 50 to 80 repetitions per minute cannot be maintained; or (v) the person says they can do no more. Our effectiveness indicator is the proportion of people making a 10% change in VO_2peak_ at the end of the 12 month intervention.

In addition, each person will also complete, the Modified Canadian Aerobic Fitness Test (mCAFT) [81], which is a graded step test and can predict VO_2peak_ using the regression equation we have recently published [46]. This test will be completed only at interim evaluations (3, 6, and 18 months) or if the person misses one of the VO_2peak_ assessments.

### Secondary outomes

A second primary effectiveness outcome will be a global test based on creating binary response variables for all relevant outcomes using known clinically meaningful change to indicate response (or ½ SD if this is not known [82]) (see Table 1).

Adherence and exercise engagement will be monitored using a combination of exercise diaries and accelerometers. The exercise diaries are a good short-term method and also serve as a way for the participants to track their progress as well as any symptom changes (positive or negative) such as fatigue or pain. We will use daily diaries for the first 3 months and subsequently ask people to complete a diary and wear an accelerometer for one of the weeks in the month preceding their scheduled assessment. The weeks for the diary and for the accelerometer will not coincide.

### Sample size

Sample size is based on a test of proportions. Assuming a range of response proportions in the General Guideline Group of 0.2 to 0.5, we have powered this study at 80% to detect a relative risk of >1.5 in favour of the MSTEP Group (Type I error 0.05). A sample size of 120 per group (total sample size of 240) is targeted.

### Randomization

Randomization will be stratified by site, Montreal and Toronto and persons will be randomly assigned, within their strata, either to the MSTEP or General Guidelines using blocked randomization. The block sizes will be 2, 4 and 6 and the size of the block will also be randomly assigned. The randomization will be done using web based program < <http://www.randomization.com.> > by a statistician who will be the only person with access to the code. When the outcome assessments have been completed by the assessment staff, the statistician will reveal the group assignment to the intervention staff.

Since funding was secured for this trial, there has been increasing interest in prescribing Fampyra to increase gait speed and thus an additional strata was created to avoid having an imbalance of people on Fampyra (dalfampridine;prolonged-release fampridine tablets) in one of the groups [83,84].

### Blinding

It is not possible to blind the participants as to their group assignment. Participants were informed that we were comparing two exercise programs and one was not presented as potentially superior to the other, protecting against bias in responses to the self-report outcomes.

Evaluators will be blinded. The main outcome is based on an assessment of VO_2peak_ which has standard procedures for administration to ensure that the subject puts as much effort as possible into the test. The test is done off-site using existing personnel who may or may not be the same for each subject or each assessment within subject. It is not a test that is strongly affected by unblinding of the testing staff. Nevertheless, the testing staff will not be informed of the group assignment. The situation for the other assessments is similar as they are performance based tests. These will be done by an assessment team of students who will vary over time but also will not be told of the group assignment.

During the analysis, the code for the group assignment is not revealed until all analyses are completed and validated.

### Statistical methods

The main analysis will be logistic regression to test the main hypothesis related to the superiority of the MSTEP program based on a greater proportion of people making a clinically relevant gain in exercise capacity at 1 year. A secondary outcome will be the differences in proportions at 2 years also using logistic regression. The analysis will be based on intention-to-treat and all persons will be analysed in the groups to which they were randomized.

A secondary analysis will estimate the impact of exercise on the other relevant outcomes. For this approach, each outcome will be converted to a binary response variable based on published clinically meaningful changes (see Table 1) and generalized estimating equations (GEE) will be used to test the rate of response in the MSTEP program to the rate of response in the general guideline approach [85,86]. If there is a statistically significant effect of the intervention, then and only then, can the effects of the separate outcomes be interpreted as real [86].

The role of exercise engagement in explaining outcome variation will be estimated using multivariate modeling for both logistic and longitudinal growth models. A separate analysis will be conducted to examine relapse rate and other adverse events. All of these analyses will be adjusted for confounders using propensity scoring [27,87-89]. Factors used in the propensity score are: extent of complaints (co-ordination, weakness or heaviness in legs, anxiety/depression, bladder problems), presence of children, and exercise enjoyment at study entry [44]. Adjustment will also include age and sex and additional prognostic variables which improve model fit.

If people are unable to do the VO_2_peak test or the data from this test is aberrant (person did not reach a peak), we will use a validated a regression equation to estimate VO_2_peak measured in ml/kg/min from submaximal tests [46]. The equation, which is based on our pilot work on 60 subjects, is: VO_2peak_ = -11.83 + 1.78(Men) + 0. 2*each 10 meters distance walked in 6 minutes + 16.3 * L/min oxygen consumption estimated from the completed stage of step test.

As is consistent with policy from the funding agency, Canadian Institutes of Health Research (CIHR), we will conduct a gender-based analysis for exploratory purposes only. We will also explore whether people with different relapsing remitting type respond differently than people with progressive type of MS. This is accomplished by fitting an interaction term between group and gender and group and type of MS. A second sub-group analysis will explore the impact of the intervention among people with a different propensity to exercise. Propensity scoring permits exploration of this variable on outcomes through stratification by low, medium, and high values on propensity for exercise interest.

To minimize potential bias arising from missing data from missing assessments or losses to follow-up, multiple imputation [90,91] will be carried out on the longitudinal data for all outcomes with sufficient data. Imputation will be based on the data arising from key measured variables and values on the health questionnaires. As is usual, 80 imputed data sets are generated and 20 are chosen (to maximize dataset independence). Multiple imputation provides estimates of the value on a missing variable that would have been recorded if the person had been assessed. The estimated values incorporate the data that are available, cross-sectionally and over time, as well as variation in the multivariate distribution of this existing data. In the analysis, both the estimate and the associated error, within and between imputed data sets, are used and the model error term thus includes the usual sources of error as well as error arising from imputation. Without this process, the p-value tends to be underestimated and more likely to cross the conventional threshold for significance [90,91].

## Discussion

The MS community is looking for interventions to help alleviate the disabling sequelae of MS. Exercise is a well-known intervention which has known benefits to all, it has no negative side effects when prescribed appropriately, it has other benefits beyond disability such as reducing obesity and cardiovascular risk. It is hypothesized to have neurorehabiliative effects [15] and it is an intervention that is accessible to all at little cost. Yet, few exercise regularly. For people with MS, our survey of exercise barriers [44] showed that a major barrier was in not knowing what to do, being afraid of heat exhaustion, and fatigue. Proper instruction and tailoring exercise to specific needs could overcome these barriers.

The exercise program is novel. It incorporates all elements of the “Activity Pyramid” [92]: lifestyle activity; aerobic activity, strength and flexibility. It incorporates the notion of a “push-day” when persons are encouraged to push themselves to exercise intensely for short bursts. It is designed to add some form of exercise to most days.

In addition, this trial incorporates a number of statistical approaches rarely applied in the rehabilitation field, yet this field typically conducts research that would benefit from more modern statistical and methodological approaches.

The primary outcome is binary not a difference of two means. This is a more interpretable outcome and facilitates knowledge translation as people with MS and their care providers can readily understand what proportion of people made a relevant gain, whereas a mean difference may not apply to individual subjects. This outcome permits a calculation of number-needed-to-treat (NNT) [93], a very useful statistic in terms on comparative effectiveness [94]. With this outcome, it is also possible for research results to present data showing factors increasing the probability of a positive response permitting participants and providers alike to identify whether an individual is in a group more or less likely to benefit and perhaps provide additional interventions to increase probability of response.

All secondary outcomes will be analysed using a global response statistic. A challenge with trials of complex interventions particularly those which tailor the intervention to patient needs, which typify rehabilitation-type interventions, is that no one outcome is likely to capture the effect equally for each person and analysing each outcome separately is often considered suspect in some research cultures. Recently in a 2009 publication in the Archives of Physical Medicine and Rehabilitation, Bagiella [85] demonstrates the value of using modern statistical methods to combine multiple outcomes into a composite response variable. We have demonstrated the benefit of this approach in evaluating a case-management intervention for people discharged home post-stroke [95].

We will also use propensity scoring, a widely used epidemiologic method to improve validity and precision when there is a need to consider multiple confounders that also may be correlated; a propensity score is calculated for each person based on their probability of taking an action or treatment, here engaging in exercise. Using this method, the propensity score is the adjustment variable, either as a continuous variable or a categorical variable such as quintiles (depending on linearity). The advantage is that multiple confounders can be combined in a single propensity score; adjustment in the analysis for the single propensity score also adjusts for all the confounders summarized in the score [89].

The results of the trial will contribute needed evidence for the development of guidelines for exercise for MS. If proven effective, the content will be made available at no cost.

## Competing interests

The authors declare that they have no competing interests.

## Authors’ contributions

NEM [Physical Therapy, Epidemiology/Biostatistics] Conceived of the idea, the research protocol, the methodology, and statistical approach; conducted all of the preliminary research supporting the science and feasibility of the trial; wrote the grant application and this protocol submission. MB [Physiatrist] Read and suggested improvements of drafts of the protocol submitted for funding; agreed to have the study conducted at the site where he is medical director; facilitated the submission of grant for ethical approval; participated in training of staff. PD [MS Neurologist] Worked on the preliminary research supporting the science and feasibility of the trial; read and suggested improvements of drafts of the protocol submitted for funding; facilitated the submission of grant for ethical approval and access to MS clinic database. YL [MS Neurologist] Worked on the preliminary research supporting the science and feasibility of the trial; read and suggested improvements of drafts of the protocol submitted for funding; facilitated the submission of grant for ethical approval and access to MS clinic database. RA [Kinesiology] Worked on the preliminary research supporting the science and feasibility of the trial; read and suggested improvements of drafts of the protocol submitted for funding; provided expertise on exercise training and testing to be conducted in his laboratory. SB [Psychology] Worked on the preliminary research supporting the science and feasibility of the trial; read and suggested improvements of drafts of the protocol submitted for funding; provided expertise on behavioural aspects of trial. All authors read and approved the final manuscript.

## Pre-publication history

The pre-publication history for this paper can be accessed here:

http://www.biomedcentral.com/1471-2377/13/69/prepub
